# Epidermal growth factor (EGF) withdrawal masks gene expression differences in the study of pituitary adenylate cyclase-activating polypeptide (PACAP) activation of primary neural stem cell proliferation

**DOI:** 10.1186/1471-2202-6-55

**Published:** 2005-08-28

**Authors:** Maria Sievertzon, Valtteri Wirta, Alex Mercer, Jonas Frisén, Joakim Lundeberg

**Affiliations:** 1 Royal Institute of Technology, AlbaNova University Center, KTH Genome Center, Dept. of Biotechnology, S-106 91 Stockholm, Sweden; 2 NeuroNova AB, S-114 33 Stockholm, Sweden; 3 Department of Cell and Molecular Biology, Medical Nobel Institute, Karolinska Institute, S-171 77 Stockholm, Sweden

## Abstract

**Background:**

The recently discovered adult neural stem cells, which maintain continuous generation of new neuronal and glial cells throughout adulthood, are a promising and expandable source of cells for use in cell replacement therapies within the central nervous system. These cells could either be induced to proliferate and differentiate endogenously, or expanded and differentiated in culture before being transplanted into the damaged site of the brain. In order to achieve these goals effective strategies to isolate, expand and differentiate neural stem cells into the desired specific phenotypes must be developed. However, little is known as yet about the factors and mechanisms influencing these processes. It has recently been reported that pituitary adenylate cyclase-activating polypeptide (PACAP) promotes neural stem cell proliferation both *in vivo *and *in vitro*.

**Results:**

We used cDNA microarrays with the aim of analysing the transcriptional changes underlying PACAP induced proliferation of neural stem cells. The primary neural stem/progenitor cells used were neurospheres, generated from the lateral ventricle wall of the adult mouse brain. The results were compared to both differentiation and proliferation controls, which revealed an unexpected and significant differential expression relating to withdrawal of epidermal growth factor (EGF) from the neurosphere growth medium. The effect of EGF removal was so pronounced that it masked the changes in gene expression patterns produced by the addition of PACAP.

**Conclusion:**

Experimental models aiming at transcriptional analysis of induced proliferation in primary neural stem cells need to take into consideration the significant effect on transcription caused by removal of EGF. Alternatively, EGF-free culture conditions need to be developed.

## Background

During the last decade it has become evident that neurogenesis occurs in certain restricted regions of the adult mammalian brain, particularly in the dentate gyrus of the hippocampus and the subventricular zone (SVZ) of the lateral ventricle [1-3]. The neurogenic cells in these areas have been isolated and can be propagated *in vitro *where they form clonal aggregates of cells denoted neurospheres [4,5]. However, there seems to be some differences between the populations of neurospheres obtained from the different regions. SVZ neurospheres possess the two cardinal properties of stem cells, i.e. they are self-renewable and multipotent [5], whereas hippocampal neurospheres appear to be more precursor cell-like, in that they are more limited in their self-renewal and individual spheres exclusively give rise to neurons or glia [6].

The rather recently discovered neural stem cell (NSC) population, sustaining continuous neurogenesis in the adult brain, has become a promising source of cells for cell-replacement therapies for various neurological diseases [7-9]. Cell replacement with fetal mesencephalic or striatal tissue has previously been shown to lead to functional improvement in patients with Parkinson's disease and Huntington's disease [10-12]. However, the use of fetal cells is hampered by a number of hurdles, in addition to ethical concerns. Fetal tissue is only available in limited quantities and the fetal cells are mostly postmitotic and cannot be expanded, nor stored for long periods of time. Furthermore these cell populations are heterogeneous, and their purity and viability cannot be reliably controlled, perhaps explaining the variation in functional outcome between different transplantation studies. In contrast, stem cells represent a source of cells that is more readily obtainable, expandable and could potentially be maintained as a more homogeneous, pure cell population. By supplying the appropriate factors endogenous stem/progenitor cells could be recruited to generate new neural or glial cells of specific phenotypes (e.g. dopaminergic neurons in Parkinson's disease or oligodendrocytes in multiple sclerosis). Alternatively the NSCs could be expanded and properly manipulated *in vitro*, before transplanting them into the appropriate area of the brain. Both strategies have been evaluated with limited but promising success [13-16]. However, more knowledge on the control of NSC proliferation and differentiation into specific phenotypes, both *in vivo *and *in vitro*, is needed before any clinical trails can be made.

Some factors stimulating neurogenesis are already known (for reviews see [7,8,17]). These include epidermal growth factor (Egf), basic fibroblast growth factor (Fgf2), brain-derived neurotrophic factor (Bdnf) and transforming growth factor-α (Tgfa). Recently there was a report that neurogenesis can also be stimulated through a G-protein coupled receptor [18]. The pleiotropic neuropeptide pituitary adenylate cyclase-activating polypeptide (PACAP) promotes NSC proliferation both *in vivo *and *in vitro*, via the PACAP receptor 1 (PAC1). PACAP belongs to the vasoactive intestinal peptide (VIP)/secretin/glucagon family of peptides and exerts a number of biological effects in addition to neurogenesis (for reviews see [19,20]).

We have recently performed a gene expression analysis of primary neural stem cells (neurospheres), demonstrating the need to consider biological fluctuations of these cells when performing comparative transcriptome analysis [21]. Here we continue the work to investigate the transcriptional changes underlying the proliferative effect of PACAP on SVZ neurospheres by using microarrays, including controls for differentiation and proliferation.

## Results

**Figure 1:**
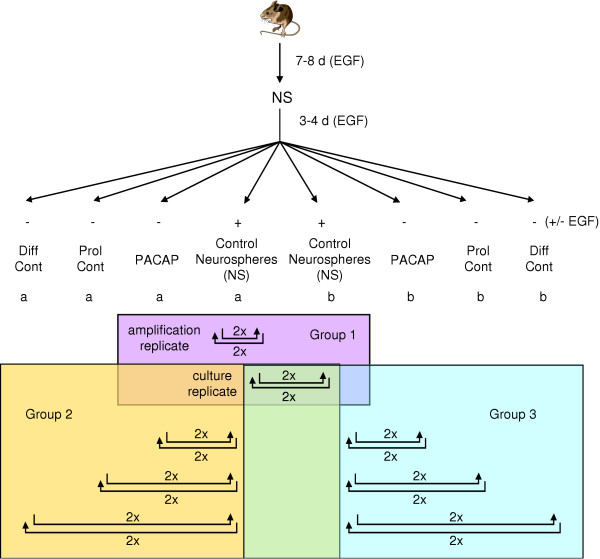
**Neurosphere culturing and experimental design**. Neural stem/progenitor cells were isolated from the lateral ventricle wall region of brains from a pool of mice and grown as neurospheres. RNA was isolated from different treatments as indicated and used for subsequent microarray hybridisations. a and b indicate biological replicates. Each arrow represents the number of hybridisations, arrowhead represents labelling with Cy5 and arrow tail represents labelling with Cy3. Groups 1, 2 and 3 indicate hybridisations grouped in the data analysis, to optimise the variance estimates for each gene. NS = neurosphere control, PACAP = pituitary adenylate cyclase-activating polypeptide treated samples, Prol Cont = proliferation control (transmembrane receptor agonist treated samples), Diff Cont = differentiation control (fetal calf serum treated, and solid support plated samples).

To investigate the molecular changes underlying the proliferative effects of PACAP we performed a gene expression analysis using a 16 k cDNA microarray. The experimental design depicted in Figure 1 was utilised. NSCs were isolated from a pool of lateral ventricular wall tissue from 15 mice and grown in culture as neurospheres, after which they were dissociated and cultured to form secondary neurospheres. Secondary neurospheres were dissociated and split into eight parallel cultures. The cells were used to study the culture variance and three different treatments, each replicated in two individual cultures (indicated by letters a and b in Figure 1.) We used amplification and culture replicates to control for technical and biological variation respectively, and controls for differentiation and proliferation activation to verify our results.

In all comparisons undifferentiated neurospheres (NS), maintained in culture medium supplemented with epidermal growth factor (EGF), was used as a reference control sample. In the first of three treatment regimes neurospheres were induced to proliferate in response to PACAP by replacing the EGF supplemented culture medium with medium supplemented with PACAP. In the proliferation control, the cells were induced to proliferate in response to a transmembrane receptor agonist (TMR agonist) by replacing EGF with the agonist in the neurosphere culture medium. In the third treatment, the differentiation control, neurospheres were induced to differentiate into neurons, astrocytes and oligodendrocytes by replacing the EGF supplemented culture medium with medium supplemented with fetal calf serum, and plating the cells onto poly-D-lysine plates.

mRNA was isolated from all cell cultures and used in a series of cDNA microarray hybridisations. To avoid extensive passaging of the neurospheres, a limited amount of cells were obtained, and the generated RNA was not sufficient for labelling and subsequent microarray analysis. The RNA was therefore amplified using a previously described protocol [22,23], that has recently also been evaluated for neurosphere analysis [21]. The principle relies on incorporating a biotin moiety into the cDNA during the first-strand cDNA synthesis, by using a biotinylated oligo(dT) primer. The population of cDNAs is fragmented and the biotinylated 3'ends captured onto a streptavidin-coated solid support. The isolated cDNA tags are released from the support, amplified by PCR and labelled for subsequent microarray hybridisation.

Amplified and labelled cDNA from each treated sample was hybridised against the neurosphere control sample (NS). In order to measure the technical variation self-to-self hybridisations were made with material from NS sample a. In addition, to measure the variation between two identical cultures, the two NS replicates (a and b) were hybridised against each other. For each comparison two replicate and two dye-swap hybridisations were performed.

We have previously shown that neurosphere culture passaging or prolonged culturing per se, is sufficient to induce differential expression and that this should be taken into account in the design of the experiment [21]. To address these issues, i.e. to get a variance measure from as many slides as possible, the results from all hybridisations were divided into three groups prior to data analysis, as indicated in Figure 1. Group 1 contains eight hybridisations comparing the technical amplification replicates (NS a-NS a) and the biological culturing replicates (NS a-NS b). Groups 2 and 3 consist of sixteen hybridisations each and include the NS a-NS b hybridisations as well as one replicate of each treatment comparison (NS vs. PACAP treated, NS vs. differentiation control and NS vs. proliferation control). This scheme allows for estimation of technical and biological noise (Group 1). Also, by using the NS a and NS b samples as reference samples in each group, the "contrasts" (i.e. the calculated differences between two treatments) can be calculated and compared between the groups (data not shown). The data in all three groups was filtered (for details see Methods) and print-tip lowess normalised using identical criteria. For each group individual gene-wise variances were calculated, and taken into account in the identification of differentially expressed genes using the empirical Bayes moderated t-test [24-26]. For each comparison the log-odds ratio (B-value) was used to rank the genes in order of evidence for differential expression. Higher B-value indicates higher probability of differential expression.

**Figure 2:**
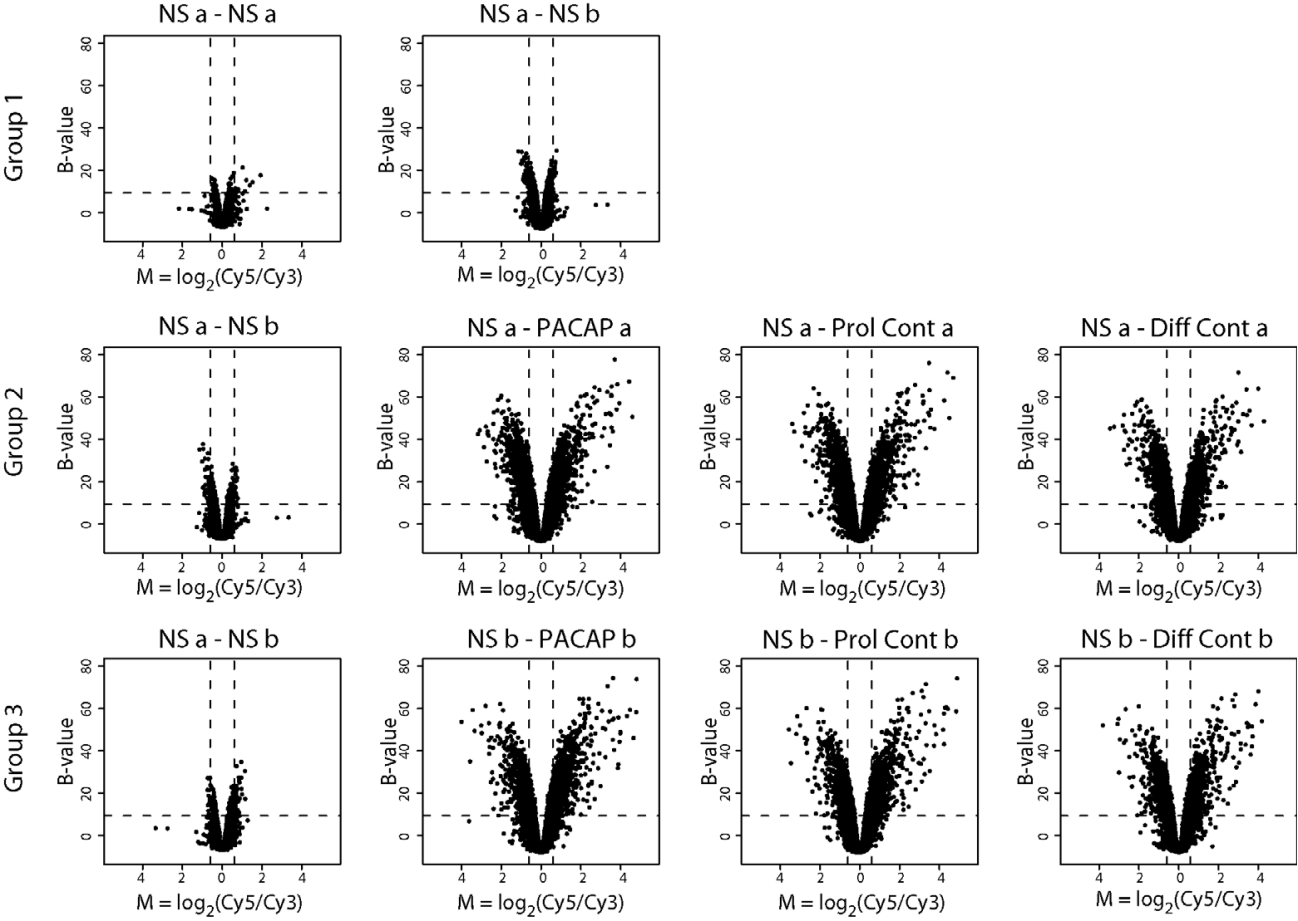
**B-value distributions for each comparison**. The x-axis shows the M-value (log_2_(Cy5/Cy3)) for each gene and the y-axis the corresponding B-value (calculated by an empirical Bayes moderated t-test).The B-value scores the genes according to their probability of differential expression. Higher B-value means higher probability of differential expression. Dotted lines are drawn at M-values 0.6 and -0.6, i.e. at a 1.5-fold difference in signal intensity between the compared samples, and at B = 9.3, corresponding to a Holm adjusted p-value of 0.0001. These values correspond to the thresholds set for differential expression in this study.

**Figure 3:**
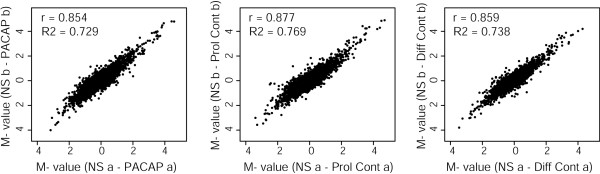
**Graphs displaying the correlation between replicated samples**. The x-axis and y-axis display M-values (log_2_(Cy5/Cy3)) for replicated samples. The values of the Pearson correlation coefficient (r) and the coefficient of determination (R^2^) are also included.

**Figure 4:**
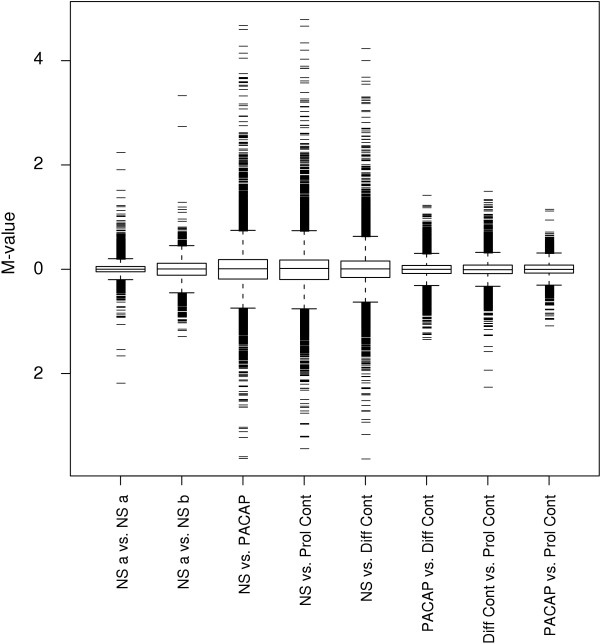
Box plots displaying the M-value (log_2_(Cy5/Cy3)) distribution for each comparison.

In order to investigate the magnitude of differential expression in each comparison the M-value (log_2_(sample X intensity/sample Y intensity)) for each gene was compared to the corresponding B-value (Figure 2). Genes with a high M-value usually receive a high B-value, which gives the plots the characteristic volcano shape. Figure 2 shows that the number of differentially expressed (DE) genes and the magnitude of differential expression is much lower for the technical and biological replicates than for the treated samples. This indicates that the RNA amplification and the biological fluctuations during culturing do not contribute substantially to the observed differential expression for PACAP and control treatments. Noteworthy, the distributions of B- and M-values are very similar for all treated samples, implicating that the magnitude of gene expression changes are similar for all treatments. The correlation between the replicated comparisons in Groups 2 and 3 was investigated by visualising the M-values from a comparison in Group 2 against the corresponding M-values in Group 3 (Figure 3). This shows that there is a high correlation between the replicates, with correlation coefficients ranging from 0.85 to 0.88. For the A-values (intensity values) the correlations are even higher ranging from 0.996 to 0.997 (data not shown). The M-value correlations between the contrasts were analysed in a similar fashion, but yielded much lower correlation coefficients (0.10 for PACAP treatment vs. proliferation control, 0.33 for differentiation control vs. proliferation control and 0.56 for differentiation control vs. PACAP treatment, data not shown). These low correlations are a consequence of the small differences between the contrasts as shown in the M-value distributions for the different comparisons (figure 4)

**Figure 5:**
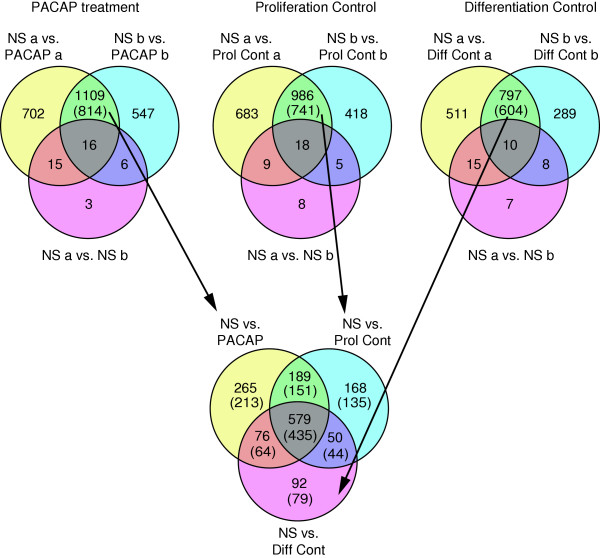
**Correlation between biological replicates and between treatments**. The number of differentially expressed genes in each comparison are presented and compared. Genes with an M-value > | 0.6 | and a p-value < 0.0001, calculated by empirical Bayes moderated t-test and a Holm's adjustment for multiple testing are included. Figures without parentheses show the number of probes included. Figures within parentheses show the number of corresponding genes. NS = neurosphere control, PACAP = pituitary adenylate cyclase-activating polypeptide treated samples, Prol Cont = proliferation control (transmembrane receptor agonist treated samples), Diff Cont = differentiation control (fetal calf serum treated, and solid support plated samples).

To further explore the overlap between DE genes in the replicated treatments Venn diagrams shown in Figure 5 were created. The comparisons include genes with a Holm adjusted p-value < 0.0001 and an M-value > +/- 0.6 (corresponding to a fold change > 1.5). Also included in the comparison are 29 DE genes (corresponding to 40 redundant probes on array) identified in the NS a-NS b comparison that could be considered as technical noise. Figure 5 shows that the overlap between the biological replicates is high. For all three treatments the majority of the genes (60–70%) are differentially expressed in both replicates; 814 genes (1109 probes) for the NS vs. PACAP treatment, 741 genes (986 probes) for the NS vs. proliferation control and 604 genes (797 probes) for the NS vs. differentiation control. Also, a large proportion of the non-overlapping genes showed a similar M-value in the two groups, indicating that the true overlap is even higher. The genes shared by both replicates for a certain treatment were further compared between the different treatments to visualise the effects of the different treatments on gene expression levels. Surprisingly, the great majority of the genes (435, 579 probes) fall within the overlap of all treatments, further suggesting that the different stimuli results in very similar effects on gene expression level.

**Figure 6:**
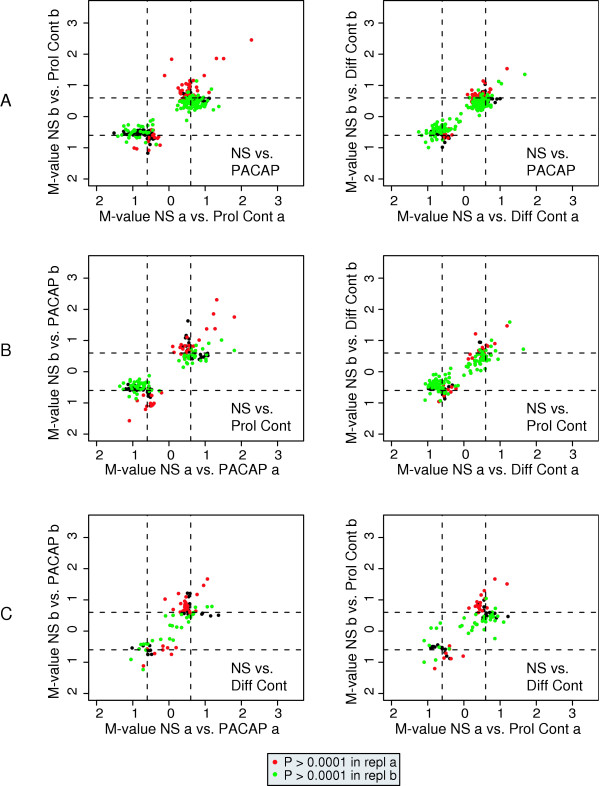
**M-value analysis of non-overlaping genes from the treatment comparisons**. M-values (log_2_(Cy5/Cy3)) for genes identified as DE in either only neurosphere vs. PACAP treatment (A), neurosphere vs. proliferation control (B) or neurosphere vs. differentation control (C) are shown using data from the other two treatment comparisons. Colouring indicates if a gene reaches the statistical significance required for differential expression (black, both replicates have p-values < 0.0001; red, p-value in replicate a > 0.0001: green, p-value in replicate b > 0.0001). Dotted lines depict the cut-off values for scoring differential expression (M-value > | 0.6 |).

A likely explanation is that the removal of growth factor (EGF) from the neurosphere culture medium, coinciding with the treatment initiation, masks the effect on gene expression changes caused by the different stimuli. To further investigate whether the remaining unique DE genes were true differences or related to the EGF effect we performed additional analysis of the 213 (265 probes), 135 (168 probes) and 79 (92 probes), non-overlapping genes. A comparison was made by taking the lists of the unique genes for one treatment and visualising their corresponding M-values in the other treatments (Figure 6). The results depicted in Figure 6 demonstrate that the majority of genes are clustered around the threshold values of the criteria for differential expression, either with an M-value just above or below 0.6 or with a p-value greater than 0.0001. Thus, the majority of unique (non-overlapping) genes are borderline cases, nearly included in the category of overlapping genes. Genes that would have been truly unique to the treatment in question would have had M-values in both replicates that were centred round zero. No such genes can be found when comparing the results from NS vs. PACAP treatment and NS vs. proliferation control. A few genes can be found when comparing NS vs. differentiation control to either one of the other two treatments, indicating that the serum treatment gives a somewhat more different gene expression profile, as expected. To facilitate further analysis of the results, annotated gene lists corresponding to the genes that were considered differentially expressed only in the NS vs. Diff Cont comparison, or shared between NS vs. PACAP and NS vs. Prol Cont comparisons are provided [see additional file 1 and additional file 2, respectively]. The complete results for all comparisons are available in ArrayExpress using experiment accession number E-MEXP-322.

These findings indicate that the differentially expressed genes in the different treatments are due to the withdrawal of EGF rather than to the treatment itself. A list of the 435 genes identified as the EGF treatment/withdrawal genes (see Figure 5) is provided [see additional file 3]. A short version of the list, with the top 40 genes, is shown in Table 1. The genes were further grouped and ranked according to their Gene Ontology annotation. We focused on the 'Biological Processes' branch of the GO theme structure and analysed the functional categories represented in the data. In total, 241 genes received a functional annotation. The results of the analysis, which was carried out at the detail level 3 (intermediate level that gives a general overview of the data), are provided [see additional file 4]. The themes with the highest representation were 'cell growth and/or maintenance' (118 genes), 'nucleobase, nucleoside, nucleotide and nucleic acid metabolism' (65), 'protein metabolism' (46), 'signal transduction' (29), 'catabolism' (20) and 'organogenesis' (20). In general, the list of expressed functional categories is enriched for various metabolism-related themes, but further down also contains themes such as 'cell adhesion', 'cell death', 'response to external stimulus' and 'cell-cell signalling' Next we analysed the overrepresentation of functional categories by using the genes represented on the array as background for the significance calculations. The top GO terms of the biological process class overrepresented in the data are shown in Table 2. A large proportion of these GO themes are related to the cell cycle and/or DNA replication, as expected for EGF-related effects. Many of the transcripts found and classified within "mitotic cell cycle" and "DNA replication and chromosome cycle" are down-regulated in the treated samples lacking EGF. In contrast many genes within "neurogenesis", "organogenesis" and "development" are up-regulated in these samples, probably reflecting the removal of inhibitory regulation on differentiation as EGF is withdrawn from the culture medium.

## Discussion

We have recently performed a pilot study investigating both the amplification technology and various neurosphere isolation and culture conditions [21]. In that study we could show a low technical variability in the microarray analysis, large transcriptional differences between passages of neurospheres and a smaller differences between parallel cultures. The variability is addressed in this study by increasing the number of replicates, using parallel cultures to improve the statistical power in identification of differentially expressed genes. The observed differential gene expression (Figure 2) is low for the culture and technical replicates, confirming previous findings, and has a minor impact on the results obtained in the three treatment comparisons.

The primary goal in this study was to identify the genes activated by PACAP treatment. PACAP acts as a neurotransmitter or neuromodulator in the brain, and regulates the secretion of certain neurohormones. PACAP also acts as a neurotrophic factor during brain development and as a neuroprotective agent in the adult brain and acts via three G-protein coupled receptors, PAC1 and the vasoactive intestinal peptide receptors VIP1 and VIP2. In the recent study [18] it was shown that PAC1 is expressed in the neurogenic regions of the adult mouse brain and in neurospheres generated from the lateral ventricular wall in the adult mouse. Importantly it was also shown that PACAP stimulates proliferation of NSC in the brain and of cultured neurospheres, and a number of new proliferation targets were sought to be discovered by the array analysis of neurospheres. The previous study [18] also demonstrated a synergistic proliferative effect of the combined stimulation of EGF and PACAP. It has previously been described that PAC1 signalling shows cross-talk to the signalling of receptor tyrosine kinase cascades, such as that elicited by nerve growth factor (NGF), suggesting that PACAP may act in concert with other molecules to maximize or regulate their effect [27]. In order to investigate the PACAP effect without EGF interference, we chose to culture the neurospheres for three days in the absence of EGF, but presence of PACAP.

However, the obtained results could not demonstrate a PACAP-specific transcriptional signature. Instead, by comparing the differentially expressed genes from PACAP treatment with control experiments representing proliferation or differentiation activation we could conclude that the removal of EGF was the main contributor to the observed differential expression. We demonstrate this by the large overlap in the Venn diagram of DE genes and we could also conclude that the group of "unique" (non-overlapping) genes were not truly unique by demonstrating that these would have been shared among the different treatments if slightly less stringent criteria had been used in the definition of differential expression. So why does EGF removal have such a strong effect in comparison to the addition of other factors, such as PACAP? EGF is an extremely powerful mitogen for adult mouse neural stem cells, exhibiting a four-fold greater proliferative potency than PACAP at 1 nM and 100 nM, respectively [18]. Such a profound mitogenic effect as that elicited by EGF is likely to require large alterations in levels of gene transcription and consequently the removal of this agent necessitates large reversals. PACAP, being a substantially less powerful proliferative agent, may well trigger less dramatic transcriptional changes, be it on genes in common (such as through the EGF pathway system) or not to those utilized by EGF to promote mitogenesis. In essence, it appears that transcriptional fluctuations induced by the presence and absence of EGF dwarf that elicited by PACAP, and indeed the TMR agonist and even differentiating factors.

Consequently, the shared DE genes reflect the removal of the mitogenic signal of EGF. Gene annotation analysis also supports the conclusions by demonstrating a down-regulation of proliferation signals and up-regulation of differentiation signals in the treated neurosphere cultures. Some of the genes observed in the differentiation control are however unique, which probably reflects a broader activation spectrum induced by fetal calf serum and solid support plating, as compared to EGF withdrawal. Previous studies of neurospheres using microarrays have also used culture conditions with withdrawal of growth factors to study differentiation [28-30]. These showed decreased expression of cell cycle related genes as well as increased expression of differentiation markers as a response to the respective treatments.

## Conclusion

In conclusion, we report that using the current study design, EGF removal in neurosphere culture medium creates too large transcriptional changes to be able to identify other parallel transcriptional events relating to proliferation by PACAP. This requires future development of modified culture conditions and assay designs to enable monitoring and proof of these transcriptional alterations.

## Methods

### Adult mouse neural stem cell culture

Adult mouse neural stem cell cultures were initiated originating from tissue isolated from 15 mice using identical dissection, dissociation and culture protocols. Briefly, the lateral wall of the lateral ventricle of 6 week-old mice was enzymatically dissociated in 0.8 mg/ml hyaluronidase and 0.5 mg/ml trypsin in Dulbecco's modified Eagle medium (DMEM) containing 4.5 mg/ml glucose and 80 U/ml DNase at 37°C for 20 min. The cells were gently triturated and mixed with three volumes of neurosphere medium (DMEM/F12, B27 supplement, 12.5 mM HEPES pH7.4) containing 20 ng/ml EGF, 100 U/ml penicillin and 100 μg/ml streptomycin. After passing through a 70-μm strainer, the cells were pelleted at 160 × g for 5 min. The supernatant was subsequently removed and the cells resuspended in neurosphere medium supplemented as above, plated in uncoated culture dishes and incubated at 37°C. Neurospheres were ready to be split 7–8 days after plating.

To split neurosphere cultures, neurospheres were collected by centrifugation at 160 × g for 5 min. The neurospheres were resuspended in 0.5 ml Trypsin/EDTA in HBSS (1×), incubated at 37°C for 2 min and triturated gently to aid dissociation. Following a further three-min incubation at 37°C and trituration, 3 volumes of ice-cold neurosphere medium containing EGF were added. The cells were pelleted at 220 × g for 4 min, resuspended in fresh neurosphere medium supplemented with 3 nM EGF.

Dissociated cells were plated and grown in neurosphere medium supplemented with EGF for a further 3–4 days by which time secondary neurospheres had developed. Secondary neurospheres were dissociated and divided into a 4 duplicated fractions. The culture control, proliferation control and PACAP-treated cultures were grown in neurosphere medium supplemented with 3 nM EGF, 1 nM transmembrane receptor agonist and 100 nM PACAP, respectively, for 3 days, and harvested for mRNA isolation. The differentiation control was replated in neurosphere medium supplemented with 1% fetal calf serum (FCS) onto poly-D-lysine plates to which the cells adhered. After incubating, overnight FCS concentration was reduced to 0.5%, and the cells cultured a further 2 days before centrifugation and subsequent mRNA isolation. All experiments were approved by the Karolinska Institute Ethical Committee.

### cDNA synthesis

Messenger RNA was isolated using Dynabeads^® ^mRNA DIRECT™ Kit from Dynal (Dynal A.S., Norway). First and RNaseH-dependent second-strand cDNA synthesis (SuperScript Choice System for cDNA Synthesis) was performed according to the manufacturer's instructions (Invitrogen, Carlsbad, CA, USA) using 45 pmol biotinylated *Not*I-oligo(dT) primer (5'-biotin-GAGGTGCCAACCGCGGCCGC (T)_15_-3'). The cDNA was phenol-chloroform extracted and ethanol precipitated and the pellet was dissolved in 40 μl of 1× TE (10 mM Tris-HCl, 1 mM EDTA). Excess *Not*I-oligo(dT) primer was removed by Chromaspinn TE-100 column (Clontech, CA, USA) purification.

### Amplification of 3'-end signature tags

The cDNA was fragmented and amplified according to a protocol previously described [22,23]. Shortly, fragmentation of the cDNA was performed in 40 μl 1× TE using an inverted sonication probe, using 16 × 10 s pulses at 90% effect (Sonifier^® ^B-12, Branson Sonic Power Company, CT, USA). Biotinylated 3'-end signature tags from the fragmented cDNA population were isolated onto 20 μl of paramagnetic streptavidin-coated beads (10 mg/ml) (Dynal A.S.) in 40 μl sample plus 40 μl Binding/Washing buffer (2 M NaCl, 0.1% Tween-20 in 1× TE, pH 7.7) at 37°C for one hour with rotation. The immobilised signature tags were end repaired using 1.5 U T4 DNA polymerase (New England BioLabs, MA, USA) in a 30-μl reaction volume at 12°C for 20 minutes according to the supplier's recommendations. Blunt-end adapters (Sima18: 5'-GGATCCGCGGTG-3'; Sima19: 5'-TCTCCAGCCTCTCACCGCGGATCC-3') were pre-annealed and ligated onto the immobilised repaired 3'-end signature tags using a solution comprising 1.1 nmol adapter, ligase buffer (66 mM Tris-HCl, pH 7.6, 5 mM MgCl_2_, 5 mM DTT, 50 μg/ml BSA), 0.2 mM ATP, 1200 U T4 DNA ligase (New England BioLabs) in a final volume of 60 μl. Ligation was performed overnight at room temperature with constant rotation to keep beads in suspension. The signature tags were released from the magnetic beads by restriction with *Not*I (New England BioLabs) for 2 hours in a volume of 60 μl while keeping the beads in suspension. Five microlitres of the eluate containing the 3'-end signature tags was used as template in a subsequent PCR. The PCR was performed in 100 μl containing 200 μM of each dNTP, 0.75 μM Sima19, 0.75 μM *Not*I-oligo(dT) primer, 65 mM Tris-HCl, pH 8.8, 4 mM MgCl_2_, 16 mM (NH_4_)_2_SO_4_, 0.5 μM BSA and 3 U AmpliTaq DNA polymerase (Perkin Elmer, Boston, USA). Cycling was performed according to the following procedure, initial incubation at 72°C for 3 min, followed by addition of *Taq *DNA polymerase and subsequent cycling: 72°C for 20 min, 95°C for 1 min, 45°C for 5 min, 72°C for 15 min, followed by four cycles (95°C for 1 min, 50°C for 1 min, 72°C for 15 min), and 13 cycles (as previously optimised) (95°C for 1 min, 50°C for 1 min, 72°C for 2 min).

### Target labelling and microarray hybridisation

The 3'-end signature tags were purified using QIAquick^® ^PCR purification kit (Qiagen, Germany). Direct labelling was performed using Cy3-dCTP or Cy5-dCTP (Perkin Elmer, Boston, USA) in a linear, asymmetric PCR. The reaction was performed in a 50-μl labelling mix containing 100–200 ng purified 3'-end signature tags, 80 μM dATP, dGTP and dTTP, 20 μM dCTP, 5 μM Sima 19 primer, 2 mM MgCl_2_, 1 × PCR Buffer II (Applied Biosystems, Ca, USA), 3 U AmpliTaq Gold^® ^(Applied Biosystems) and 60 pM Cy3-dCTP or Cy5-dCTP. The labelling mix was cycled as follows: 95°C for 12 min, then 20 cycles (95°C for 30 s, 50°C for 30 s, 72°C for 10 min). Excess primer and nucleotides were removed using QIAquick^® ^PCR purification kit. The eluted labelling products were speed vacuumed until dry, then dissolved in 55 μl hybridisation buffer (24% formamide, 5× SSC and 0.1% SDS). Cy-3 and Cy-5 labellings were blended and mixed with 25 μg mouse Cot-1 DNA (Invitrogen) and 50 μg polyA DNA (Operon Biotechnologies GmbH, Germany). The microarray contained 14121 probes printed in duplicate and originating mainly from a lateral ventricle wall (Unigene Library ID 16789), a neurosphere (Lib. 16808) and a hematopoietic cell-line cDNA (Lib. 16809) library. Details regarding the array manufacturing are available through ArrayExpress (accession number A-MEXP-175). Briefly, probes were generated through PCR amplification and subsequent Multiscreen-384 filter plates (Millipore) purification of the library clones. Purified products in 50% DMSO were printed onto ultraGAPS slides (Corning Inc) using the QArray arrayer (Genetix) and covalently attached to the slide surface using 250 mJ UV-light (Stratalinker). The arrays were first prehybridised for 30 min in a 42°C prehybridisation solution (1% BSA, 5× SSC, 0.1% SDS), then washed in water and isopropanol and dried through centrifugation. The sample was denatured in 95°C for 3 min, applied to the array and incubated in a hybridisation chamber at 42°C for 18 hours. After hybridisation the arrays were washed in three successive wash buffers with increasing stringency: (1) 1× SSC and 0.2% SDS, 42°C, (2) 0.1× SSC and 0.2% SDS, room temperature, (3) 0.1× SSC, room temperature. All wash steps were made on a shaking table for 4 min. After the last step the arrays were immediately centrifuged in a slide centrifuge and kept in the dark until scanned with the DNA Microarray scanner G2565BA (Agilent Technologies, CA, USA).

### Image and data analysis

All image and data analysis was conducted in GenePix Pro 5.1 (Axon Instruments Inc, CA, USA) and the R environment for statistical computing and programming [31] using packages aroma [32], Bioconductor [33], limma [25] and kth [34]. The analysis was conducted according to the following workflow. (1) Spot identity and foreground/background intensities were extracted from the tiff files using the irregular feature-finding algorithm implemented in GenePix Pro 5.1. (2) GenePix results files were imported into R and the median of the foreground signal was used as expression measurements without subtracting the local background signal. (3) Flagged features (either automaticly by GenePix or manually by the user), too small or large features (<51 and >250 μm, respectively), saturated features (both channels > 65100 intensity units) or features for which the signal-to-noise ratio (as defined by GenePix 5.1) was < 3, were removed from further analysis. (4) Remaining data was normalised separately for each block on the slide using the robust intensity-dependent print-tip lowess normalisation approach [35]. (5) Differentially expressed genes were identified using an empirical Bayes moderated t-test [24-26] and ranked in order of evidence for differential expression. The p-values associated with the t-test were adjusted for multiple testing by using the Holm's approach [36]. Genes with an adjusted p-value < 0.0001 were considered differentially expressed. Genes with very small fold-changes (< 1.5 fold) were excluded by using an additional M-value cut-off (> +/- 0.6). ESTs on the array were mapped against build 144 of *Mus musculus *Unigene. Over-representation analysis of functional cateogories or themes was carried out using the EASE software [37]. Themes with an EASE-score < 0.05 were considered overrepresented. The complete set of raw data and transformed data is available through ArrayExpress (experiment accession number E-MEXP-322).

## List of abbreviations

NSC; neural stem cell

SVZ; subventricular zone

DE; differentially expressed

NS; neurosphere

DC; differentiated cells

PACAP; pituitary adenylate cyclase-activating polypeptide

EGF; epidermal growth factor

## Authors' contributions

MS participated in the design of the study, drafted the manuscript, coordinated and carried out microarray experiments as well as performed data processing and data analysis. VW coordinated and carried out the manufacturing of the microarrays, performed data analysis and statistical analysis as well as assisted with the manuscript. AM participated in the design of the study, cultured cells and assisted with the manuscript. JF participated in its design and coordination and assisted with the manuscript. JL conceived of the study, participated in its design and coordination of the study and helped to draft the manuscript, principle investigator. All authors read and approved the final manuscript.

## Supplementary Material

Additional File 1 Differentially expressed genes in the NS vs. Diff Cont comparison only. Differentially expressed genes (with an M-value > | 0.6 | and a p-value < 0.0001, calculated by empirical Bayes moderated t-test and a Holm adjustment for multiple testing) in the NS vs. Differentiation Control only, corresponding to the 79 genes in Fig 5. To facilitate comparisons the M- and Holm adjusted p-values for all treatment replicates are provided. Genes that have positive M-values are up-regulated in the treated (EGF withdrawn) samples, compared to the undifferentiated neurospheres (NS). Genes that have negative M-values are up-regulated in the NS sample. Click here for file

Additional File 2 Differentially expressed genes in the NS vs. Proliferation samples only. Differentially expressed genes (with an M-value > | 0.6 | and a p-value < 0.0001, calculated by empirical Bayes moderated t-test and a Holm adjustment for multiple testing) in the overlap between the NS vs. PACAP and NS vs. Prol Cont comparisons, corresponding to the 151 genes in Fig 5. To facilitate comparisons the M- and Holm adjusted p-values for all treatment replicates are provided. Genes that have positive M-values are up-regulated in the treated (EGF withdrawn) samples, compared to the undifferentiated neurospheres (NS). Genes that have negative M-values are up-regulated in the NS sample. Click here for file

Additional File 3 Differentially expressed genes in all treatments. Differentially expressed genes in the overlap between all treatments (NS vs. PACAP, NS vs. Prol Cont and NS vs. Diff Cont). Genes that have positive M-values are up-regulated in the treated (EGF withdrawn) samples, compared to the undifferentiated neurospheres (NS). Genes that have negative M-values are up-regulated in the NS sample. Provided are the M-, B- and p-values for all treatment replicates, as well as the average M- and B-values and their standard deviations. All genes have an M-value > | 0.6 | and a p-value < 0.0001, calculated by empirical Bayes moderated t-test and a Holm adjustment for multiple testing, in all treatments. Click here for file

Additional File 4 GO theme representation among the EGF treatment/withdrawal genes. Representation of gene ontology themes among the genes that were differentially expressed in all treatments. Analysis was restricted to themes of category biological process. Genes with M-value > | 0.6 | and p < 0.0001, calculated by empirical Bayes moderated t-test and a Holm adjustment for multiple testing, are included. Click here for file

## Figures and Tables

**Table 1:** Top differentially expressed genes in the overlap between all treatments.

**Unigene ID**	**Gene ID**	**GeneName**	**Avg M**	**Stdev M**	**Avg B**	**Stdev B**
Mm.305535	69719	Carbamoyl-phosphate synthetase 2	4.53	0.26	53.33	4.34
Mm.1239	14580	Glial fibrillary acidic protein	4.47	0.39	69.36	3.97
Mm.252063	17196	Myelin basic protein	3.84	0.57	47.80	7.00
Mm.200608	12759	Clusterin	3.72	0.54	60.76	7.92
Mm.240850	76960	Breast carcinoma amplified sequence 1	3.72	0.17	46.89	1.30
Mm.291129	70877	RIKEN cDNA 4921517J23 gene	3.68	0.44	49.64	6.06
Mm.1268	18823	Proteolipid protein (myelin) 1	3.64	0.49	51.72	4.02
Mm.305152	11816	Apolipoprotein E	3.62	0.46	45.79	11.39
Mm.334994	53867	Procollagen. type V. alpha 3	3.61	0.45	49.05	8.48
Mm.272443	64383	Sirtuin 2	3.33	0.34	72.98	3.98
Mm.7420	22153	Tubulin. beta 4	3.13	0.38	53.65	4.65
Mm.39053	71770	Adaptor-related protein complex 2. beta 1 subunit	2.92	0.61	32.53	10.40
Mm.228362	NA	Transcribed locus	2.69	0.15	39.82	3.13
Mm.358573	11858	Ras homolog gene family. member N	2.68	0.39	52.12	7.88
Mm.276739	20665	SRY-box containing gene 10	2.65	0.34	33.25	1.34
Mm.241355	17136	Myelin-associated glycoprotein	2.57	0.59	32.28	6.80
Mm.278672	14810	Glutamate receptor. ionotropic. NMDA1 (zeta 1)	2.56	0.52	35.15	7.11
Mm.260601	242521	Kelch-like 9 (Drosophila)	2.54	0.41	44.19	9.26
Mm.192991	17748	Metallothionein 1	2.47	0.54	21.61	5.08
Mm.37199	14862	Glutathione S-transferase. mu 1	2.44	0.16	38.61	2.15
						
Mm.41580	69908	RAB3B. member RAS oncogene family	-3.55	0.31	47.83	4.75
Mm.290563	12615	Centromere autoantigen A	-3.14	0.16	49.02	3.58
NA	NA	GenBank ID: CX204578	-3.09	0.34	34.48	2.55
NA	NA	GenBank ID: CX207566	-2.99	0.27	51.46	6.04
Mm.273049	12443	Cyclin D1	-2.89	0.33	44.78	3.46
Mm.289747	107995	Cell division cycle 20 homolog (S. cerevisiae)	-2.68	0.15	55.41	5.67
Mm.285723	14793	Cell division cycle associated 3	-2.65	0.08	42.97	0.95
Mm.24337	52033	PDZ binding kinase	-2.58	0.12	40.31	2.70
Mm.29254	16009	Insulin-like growth factor binding protein 3	-2.49	0.29	41.37	3.97
Mm.233799	16010	Insulin-like growth factor binding protein 4	-2.46	0.31	23.86	5.98
Mm.16525	18817	Polo-like kinase 1 (Drosophila)	-2.43	0.12	26.12	1.92
Mm.28038	52276	Cell division cycle associated 8	-2.39	0.12	30.79	5.44
Mm.4857	12323	Calcium/calmodulin-dependent protein kinase II. beta	-2.38	0.49	42.14	5.06
Mm.335368	NA	CDNA. clone:Y2G0133B03:ENSEMBL	-2.35	0.25	50.92	4.35
NA	NA	GenBank ID: CX206925	-2.32	0.13	28.39	1.66
Mm.271711	21346	Transgelin 2	-2.25	0.12	38.33	1.70
Mm.4237	21973	Topoisomerase (DNA) II alpha	-2.14	0.12	46.22	2.01
Mm.1408	11535	Adrenomedullin	-2.07	0.17	26.68	5.70
Mm.37802	69544	WD repeat domain 5B	-2.03	0.10	59.04	2.30
Mm.35389	13063	Cytochrome c. somatic	-2.02	0.12	51.69	2.15

The top of the table shows genes that are up-regulated in the treated (EGF withdrawn) samples, compared to the undifferentiated neurospheres (NS) (positive M-values). The lower half of the table shows genes that are up-regulated in the NS sample (negative M-values). Average M- and B-values for all comparisons (NS vs. PACAP, NS vs. Prol Cont and NS vs. Diff Cont) are shown, together with their standard deviations. All genes have a p-value < 0.0001, calculated by empirical Bayes moderated t-test and a Holm adjustment for multiple testing.

**Table 2:** Gene ontology analysis.

**Biological process enriched in neurosphere (NS) control sample**	**No of DE genes in all treatments**	**No of genes on array**	**Fisher Exact**
mitotic cell cycle	35	136	3.840E-22
DNA replication and chromosome cycle	24	72	3.320E-18
cell cycle	43	307	2.360E-16
cell proliferation	46	362	7.620E-16
DNA replication	20	61	3.770E-15
S phase of mitotic cell cycle	20	62	5.400E-15
DNA-dependent DNA replication	11	24	1.160E-10
DNA metabolism	29	218	1.830E-10
DNA replication initiation	7	8	4.430E-10
mitosis	12	48	5.240E-08
M phase of mitotic cell cycle	12	48	5.240E-08
cell growth and/or maintenance	79	1411	2.740E-07
nuclear division	12	58	4.950E-07
M phase	12	61	8.830E-07
cellular physiological process	82	1564	2.770E-06
cytokinesis	11	63	8.760E-06
nucleosome assembly	6	21	5.920E-05
regulation of cell cycle	15	138	7.790E-05
physiological process	135	3454	6.310E-04
DNA packaging	9	80	1.750E-03
			
**Biological process enriched in treated (PACAP. Prol Cont and Diff Cont) samples**	**No of DE genes in all treatments**	**No of genes on array**	**Fisher Exact**

microtubule-based process	7	64	1.340E-03
development	22	456	3.400E-03
neurogenesis	8	99	4.300E-03
organogenesis	14	243	4.390E-03
morphogenesis	15	282	6.730E-03
cytoskeleton organization and biogenesis	9	134	8.430E-03
organelle organization and biogenesis	10	175	1.650E-02
cellular process	65	2144	6.560E-02
cytoplasm organization and biogenesis	10	222	6.720E-02
lipid metabolism	8	173	8.570E-02
membrane lipid metabolism	3	42	9.840E-02
cell adhesion	7	162	1.360E-01
cell communication	27	835	1.440E-01
cell organization and biogenesis	12	331	1.620E-01
regulation of biological process	5	110	1.650E-01
ion transport	7	188	2.280E-01
cellular physiological process	45	1564	2.640E-01
cation transport	5	135	2.850E-01
cell death	5	144	3.330E-01
cell surface receptor linked signal transduction	9	286	3.440E-01

The most highly overrepresented gene ontology themes among the genes that were differentially expressed in all treatments. Analysis was restricted to themes of category biological process. Genes with M-value > | 0.6 | and p < 0.0001, calculated by empirical Bayes moderated t-test and a Holm adjustment for multiple testing, are included. Jacknife Fisher's exact probability test was used to identify over-represented themes in the data. Corresponding p-values are listed.
